# Long-term outcomes of an educational intervention to reduce antibiotic prescribing for childhood upper respiratory tract infections in rural China: Follow-up of a cluster-randomised controlled trial

**DOI:** 10.1371/journal.pmed.1002733

**Published:** 2019-02-05

**Authors:** Xiaolin Wei, Zhitong Zhang, Joseph P. Hicks, John D. Walley, Rebecca King, James N. Newell, Jia Yin, Jun Zeng, Yan Guo, Mei Lin, Ross E. G. Upshur, Qiang Sun

**Affiliations:** 1 Division of Clinical Public Health and Institute for Health Policy, Management and Evaluation, Dalla Lana School of Public Health, University of Toronto, Toronto, Ontario, Canada; 2 China Global Health Research and Development, Shenzhen, China; 3 Nuffield Centre for International Health and Development, University of Leeds, Leeds, United Kingdom; 4 School of Health Care Management, Shandong University, Jinan, China; 5 Key Laboratory of Health Economics and Policy Research, National Health Commission, Jinan, China; 6 Guangxi Autonomous Region Centre for Disease Control and Prevention, Nanning, China; 7 School of Public Health, Peking University, Beijing, China; Massachusetts General Hospital, UNITED STATES

## Abstract

**Background:**

Inappropriate antibiotic prescribing causes widespread serious health problems. To reduce prescribing of antibiotics in Chinese primary care to children with upper respiratory tract infections (URTIs), we developed an intervention comprising clinical guidelines, monthly prescribing review meetings, doctor–patient communication skills training, and education materials for caregivers. We previously evaluated our intervention using an unblinded cluster-randomised controlled trial (cRCT) in 25 primary care facilities across two rural counties. When our trial ended at the 6-month follow-up period, we found that the intervention had reduced antibiotic prescribing for childhood URTIs by 29 percentage points (pp) (95% CI −42 to −16).

**Methods and findings:**

In this long-term follow-up study, we collected our trial outcomes from the one county (14 facilities and 1:1 cluster randomisation ratio) that had electronic records available 12 months after the trial ended, at the 18-month follow-up period. Our primary outcome was the antibiotic prescription rate (APR)—the percentage of outpatient prescriptions containing any antibiotic(s) for children aged 2 to 14 years who had a primary diagnosis of a URTI and had no other illness requiring antibiotics. We also conducted 15 in-depth interviews to understand how interventions were sustained.

In intervention facilities, the APR was 84% (1,171 out of 1,400) at baseline, 37% (515 out of 1,380) at 6 months, and 54% (2,748 out of 5,084) at 18 months, and in control facilities, it was 76% (1,063 out of 1,400), 77% (1,084 out of 1,400), and 75% (2,772 out of 3,685), respectively. After adjusting for patient and prescribing doctor covariates, compared to the baseline intervention-control difference, the difference at 6 months represented a 6-month intervention-arm reduction in the APR of −49 pp (95% CI −63 to −35; *P* < 0.0001), and compared to the baseline difference, the difference at 18 months represented an 18-month intervention-arm reduction in the APR of −36 pp (95% CI −55 to −17; *P* < 0.0001). Compared to the 6-month intervention-control difference, the difference at 18 months represented no change in the APR: 13 pp (95% CI −7 to 33; *P* = 0.21). Factors reported to sustain reductions in antibiotic prescribing included doctors’ improved knowledge and communication skills and focused prescription review meetings, whereas lack of supervision and monitoring may be associated with relapse. Key limitations were not including all clusters from the trial and not collecting returned visits or sepsis cases.

**Conclusions:**

Our intervention was associated with sustained and substantial reductions in antibiotic prescribing at the end of the intervention period and 12 months later. Our intervention may be adapted to similar resource-poor settings.

**Trial registration:**

ISRCTN registry ISRCTN14340536.

## Abstract 2

Xiaolin Wei and colleagues report long-term outcomes from their trial, which aimed to reduce prescribing of antibiotics in Chinese primary care to children with upper respiratory tract infections.

## Author summary

**Why was this study done?:**

- Antibiotic resistance is a global threat to health and effective healthcare, and a major driver of antibiotic resistance is inappropriate prescribing of antibiotics.
- In low- and middle-income countries (LMICs) such as China, huge amounts of antibiotics are inappropriately prescribed to children in primary care for upper respiratory tract infections (URTIs), such as common colds.
- However, very little evidence exists about what kinds of interventions can effectively reduce this kind of inappropriate prescribing in China and other LMICs.

**What did the researchers do and find?:**

- We developed an intervention that (1) trained rural Chinese primary care doctors to appropriately prescribe antibiotics for childhood URTIs and to better explain to patients/caregivers why they do not need antibiotics (when they do not), (2) made doctors regularly review their own and their colleagues’ antibiotic prescribing practices and provided feedback to improve appropriate prescribing, and (3) provided educational materials to patients/caregivers.
- We originally tested the intervention in 12 facilities compared to 13 facilities without the intervention and found that, after 6 months, our intervention reduced inappropriate prescribing of antibiotics for childhood URTIs by 29 percentage points (pp).
- In this study, we looked at what happened 12 months after the trial ended (and intervention facilities were not obliged to continue the intervention) in seven intervention facilities compared to seven nonintervention facilities and found that antibiotic prescribing for childhood URTIs was still 36 pp lower in the intervention facilities.

**What do these findings mean?:**

- Interventions that train and educate doctors and patients/caregivers about appropriate prescribing as well as forcing doctors to review their and their colleagues’ prescribing practices can substantially reduce inappropriate antibiotic prescribing over long timescales.
- We would recommend that practices be put in place to regularly refresh doctors’ training and ensure that prescribing reviews are maintained to avoid the effects of such interventions declining over time.
- This kind of intervention needs to be adapted and tested in other LMICs across a wide range of healthcare levels to combat antimicrobial resistance.

## Introduction

Preventing the development of antimicrobial resistance is a global policy priority. Overuse of antibiotics directly promotes development of antimicrobial resistance [1]. The largest cause of inappropriate antibiotic use comes from treating respiratory infections, which are also the most common reason for primary care consultations [2]. This challenge is most pressing in low- and middle-income countries (LMICs), where commonly 80% of upper respiratory tract infections (UTRIs), which are mostly viral, are inappropriately treated with antibiotics [3] compared to around 20% of URTIs in high-income countries (HICs) [4]. Interventions aimed at both clinicians and patients/caregivers have been shown to be more effective than those aimed at only one group [5]. Only 16 randomised trials trying to reduce inappropriate antibiotic prescribing for URTIs have been conducted worldwide, and most in LMICs had limitations in trial design and assessment. The duration of two well-designed trials in LMICs were shorter (14 days [6] and 6 months [7]) than those in HICs (generally 12 months) [8–10]. The effect sizes achieved were around a 25 percentage point (pp) reduction in antibiotic prescribing in LMICs [6,7] compared to around 10 pp in HICs [8–10], mainly because the baseline level of antibiotic prescribing was much higher in LMICs. Studies examining the sustained effects of trial interventions are rare: only two were conducted in HICs and reported sustained trial intervention effects [11,12]. In LMICs, primary care doctors are poorly trained, receive much lower pay, and see substantially more patients per day compared with their peers in HICs [13], while antimicrobial stewardship is weak [14]. Sustained evidence on interventions that work for a long term in these settings is urgently needed.

Our cluster-randomised trial was done in 25 primary care facilities known as township hospitals (with township-level divisions being the basic administrative or political divisions in rural China, typically having a population between 50,000–150,000) across two counties in rural China. It produced a 29 pp reduction in the antibiotic prescription rate (APR) among children aged 2 to 14 years old with URTIs within 6 months [7]. Our intervention targeted both doctors and caregivers. In the first month, we conducted training to improve township doctors’ knowledge on antibiotic prescribing using concise clinical guidelines. Doctors were also trained to improve their communication skills and give short educational messages to caregivers during consultations, and monthly prescribing peer-review meetings were set up. Caregivers received leaflets and watched videos on appropriate antibiotic use in waiting rooms. The intervention was designed to be self-sustaining, e.g., prescription peer reviews were first conducted with the trial team and then by each facility during their routine monthly clinician meetings. This embedded approach is believed to improve acceptability and sustainability of interventions to change clinical practice [15]; however, the long-term effect is unknown. Here, we report our trial outcomes for one county in which electronic records were available, 18 months after the start of the intervention or 12 months after the original follow-up, to evaluate whether our multifaceted intervention produced sustained outcomes in reducing antibiotic prescribing for childhood UTRIs in rural China.

## Methods

Our study is reported here as per the CONSORT extension (S1 CONSORT Checklist) for cluster trial guidelines [16], with the abstract reported as per the CONSORT guidelines for reporting randomised trials in journal and conference abstracts [17]. We obtained ethical approval from the University of Leeds School of Medicine Research Ethics Committee (MREC15-016), the Guangxi Institute Review Boards at the Guangxi Autonomous Region Centre for Disease Control and Prevention (CDC) (GXIRB2014-0036), and Shandong University (20151102) for the trial and follow-up study.

### Study design and participants

Our trial protocol and report describe the details of our trial design, intervention, and outcomes [7,18]. In summary, our trial was a pragmatic, two-arm, cluster-randomised controlled trial (cRCT) that aimed to reduce inappropriate prescribing of antibiotics for URTIs in children aged 2 to 14 years old presenting as outpatients to township hospitals in rural Guangxi, China. We used a cluster design because it was not possible to deliver the intervention at an individual level. We recruited 25 township hospitals, 14 from Rong county and 11 from Liujiang county, and randomised them all at the same time—stratified by county—using a computer programme in a 1:1 (intervention to control) ratio in Rong county and a 5:6 ratio in Liujiang county. Only the two facilities located in the county centres were ineligible. We did not originally plan to collect post-trial outcomes but decided to conduct a long-term follow-up after randomisation. However, because only Rong county had electronic prescription records available, due to logistical limitations, we restricted this follow-up study to Rong county, and all subsequent methods relate to this county alone. Within Rong county, we randomly selected six internal pilot facilities (three intervention and three control) from the 14 already recruited and randomised. We implemented the interventions in the three internal pilot intervention facilities for a 6-month period between July and December 2015 and, in the remaining four intervention facilities, between October 2015 and March 2016. After trial completion, we followed up each of the 14 facilities for 12 months between January 2016 and March 2017.

### Outcomes

Although we did not publish our planned outcomes for this long-term follow-up study via a protocol, we used the same outcomes that we used in our trial. Our primary outcome is the APR—the percentage of prescriptions given to 2- to 14-year-old outpatients for URTIs that contain any antibiotic(s). Our secondary antibiotic prescribing outcomes relate only to those prescriptions including antibiotic(s), and were the percentage of antibiotic-containing prescriptions that included (1) more than one antibiotic, (2) any broad-spectrum antibiotic(s), and (3) any intravenously administered antibiotic(s). These outcomes can therefore reflect changes in the proportion of specific types of antibiotics prescribed, as distinct from changes in the overall proportion of prescriptions containing antibiotics. Additional secondary prescribing outcomes and cost outcomes, which relate to all child URTI prescriptions, were the percentage of prescriptions containing any (4) antivirals, (5) glucocorticoids, (6) vitamins, (7) traditional Chinese medicines, and (8) any other nonantibiotic medicine(s); and (9) the full prescription cost (including the total of any consultation costs, treatment costs, and medication costs), (10) the cost of all antibiotic medications, and (11) the cost of all nonantibiotic medications. We measured all outcomes at baseline, 6-month follow-up (trial endline), and 18-month follow-up. In our trial, we added outcomes 3, 4, 5, 6, 7, 8, 10, and 11 post protocol, and a protocol-planned secondary outcome of the quinolone prescription rate was too rare in children for meaningful analysis and was excluded.

### Procedures

We ended trial activities 6 months after implementation, with the trial team stopping any direct interventions including organised training sessions or supervisory activities to monitor antibiotic prescribing. However, the intervention clusters adopted the antimicrobial stewardship programme into routine practice, which included referring to our guidelines for clinical knowledge and communication skills regarding respiratory disease, conducting peer reviews of antibiotic prescribing on a regular basis in existing team meetings, and providing concise education to patients on antibiotic use. Control clusters continued receiving usual care, in which antibiotics were given at the individual clinician’s discretion, with no peer reviews for antibiotic prescribing or caregiver education. We could not blind doctors or caregivers to treatment allocation.

### Data collection

We obtained all outcome data from electronic medical records of prescriptions issued by the 14 facilities in Rong county. Eligible prescriptions were those from children between 2 and 14 years old who had a primary diagnosis of a URTI as defined according to the International Classification of Diseases 10th Revision [19]. We excluded any child who had a secondary diagnosis of lower respiratory tract infections (such as pneumonia), who had a diagnosis of acute otitis media (for which antibiotics would be appropriate), or who had any severe and/or chronic disease requiring long-term antibiotic treatment or prophylaxis (S1 Table). For our analyses, we collected eligible outpatient prescriptions at baseline (during the 3 months prior to the start of the 6-month intervention period), at the 6-month trial endline (during the final 3 months of the 6-month intervention period), and at 18-month follow-up (during the final 3 months of the 18-month period since the intervention was first implemented). Based on the trial protocol and analysis plan, we then randomly selected 200 eligible prescriptions from each arm at baseline and 6 months and included all eligible prescriptions at 18 months for this study.

We conducted a qualitative study in three purposively selected township hospitals: two in the intervention arm (one better performing and one less well performing in the trial based on APR reductions) and one in the control arm. We did in-depth interviews with three township hospital directors (one in each hospital), six doctors (two in each hospital), and six caregivers of children (two in each hospital). We conducted interviews face to face by audio recording and by taking notes. We used a semistructured interview guide covering key areas of interests but retained the flexibility to accommodate unanticipated topics raised by participants. Our interviews covered (1) doctors’ views on intervention components and any persistent change, (2) doctors’ views on consultations with URTI patients and use of antibiotics, (3) caregivers’ knowledge and beliefs about antibiotics and any nonprescribed use, and (4) any contextual factors that affected antibiotic use (see S1 Text for interview guides). We obtained written (or verbal for illiterate caregivers) informed consent from all participants.

### Statistical analysis

Our trial’s sample size is detailed in our trial paper [7]. For this long-term follow-up study, because we were using only electronic records, we also included all available eligible prescriptions issued during the 12-month follow-up period across the facilities in Rong county in addition to using all previously analysed prescriptions. We did not publish our planned analyses for this long-term follow-up study via a protocol. However, in this study, we analysed the same outcomes and adjusted for the same covariates (apart from county given that we only used data from one county) in our adjusted analyses as used in our trial. We also based our inferences in this study on our adjusted results as in our trial (and, as in our trial, we also present covariate-unadjusted results for comparison). Compared to our trial, we had to use different methods of analysis in this study to allow us to estimate treatment effects between the different time points. However, in this study (as with our trial), we based our inferences on the same type of treatment effects (absolute differences between treatment arms for both binary and continuous outcomes).

We calculated treatment effects using generalised estimating equations (GEEs), accounting for clustering within facilities, and between time periods within facilities, via an exchangeable correlation matrix. Where the models did not converge, we employed an identity correlation matrix, given that GEEs are robust to misspecification of the correlation matrix [20]. We used time-by-treatment interactions in all models to estimate treatment effects as either (1) the difference between the intervention minus control difference at 6 months and the intervention minus control difference at baseline, (2) the difference between the treatment difference at 18 months and the treatment difference at baseline, or (3) the difference between the treatment difference at 18 months and the treatment difference at 6 months. This allowed us to understand the treatment’s effect on outcomes from baseline to the end of the trial period, from baseline to the end of the follow-up period, and from the end of trial-directed intervention activities to the end of the follow-up period. We calculated absolute treatment effects for our binary prescribing outcomes on the pp scale using GEEs with binomial errors and identity links, which have been shown to produce robust estimates of absolute treatment effects for binary clustered data [21]. Where these models failed to converge as recommended, we next tried GEEs with Poisson errors and identity links, and if they still failed, we used GEEs with Gaussian errors and identity links, which have also been shown to produce robust absolute treatment effects for binary clustered data [21]. For our cost outcomes, we used generalised linear models (GLMs) with identity links and Gaussian errors to produce estimates of mean differences [20]. We present covariate-adjusted results as our primary results because of the potential for gains in power and precision, and following our trial analyses, we adjusted models for patients’ sex, age, and payment type (insured or fully out of pocket), as well as doctors’ sex, age, and qualification level (based on 3 years of education or 5 years [MBBS equivalent]), although we did not adjust for county given that, in this study, we only had data from one county. Because diagnosis was not adjusted for in our original trial analyses but might be a confounding factor, we also did a sensitivity analysis including diagnosis as an additional covariate in the adjusted analysis of our primary outcome. We also calculated crude (unadjusted for additional covariates) model results for comparison to our adjusted results. We did not do any subgroup analyses because our trial paper subgroup analyses (looking at effect modification by patient sex and payment type, as well as clinician sex, age, qualification level, and years of experience) showed no evidence of subgroup differences in effectiveness [7]. As with our trial analyses, we did not adjust the type 1 error rate for our secondary outcome analyses and treat them as exploratory. There were no missing outcome data, but as with our trial analyses, we excluded prescriptions with missing covariate data (<1%, see S3 Table) from the adjusted analyses, which therefore assume that data are missing at random. As with our trial analyses, there were no changes to the original treatment allocations of any clusters, and therefore the crude analyses that used all data were on an ‘intention-to-treat basis’, while the adjusted analyses were on a ‘modified intention-to-treat’ basis due to the use of complete cases only where covariate data were missing. We used Stata version 14 (Stata Corporation, College Station, TX) for all analyses.

### Qualitative analysis

We transcribed interviews verbatim in Standard Chinese. We employed thematic framework analysis, with two researchers independently coding transcripts, indexing interview transcripts, and charting indexes. Two researchers (ZZ and JY) independently coded transcripts, indexed interview transcripts, and charted indexes. Any discrepancy was discussed and agreed upon unanimously. Themes were discussed extensively and agreed upon within the research team. The two bilingual researchers also translated the themes into English, and reviewed line by line. Then, the themes were double checked by the principal investigator (XW) and the qualitative study lead (RK). The analysis was guided by the theoretical domains framework (TDF) that examines factors influencing different domains such as knowledge, skills, and behaviour changes of providers and patients [18,22]. We categorised them into procedural, interpersonal, and contextual factors. The research team discussed themes extensively before agreeing on them. We used NVivo 10 (QSR International Pty Ltd.) for analysis.

## Results

**Fig 1:**
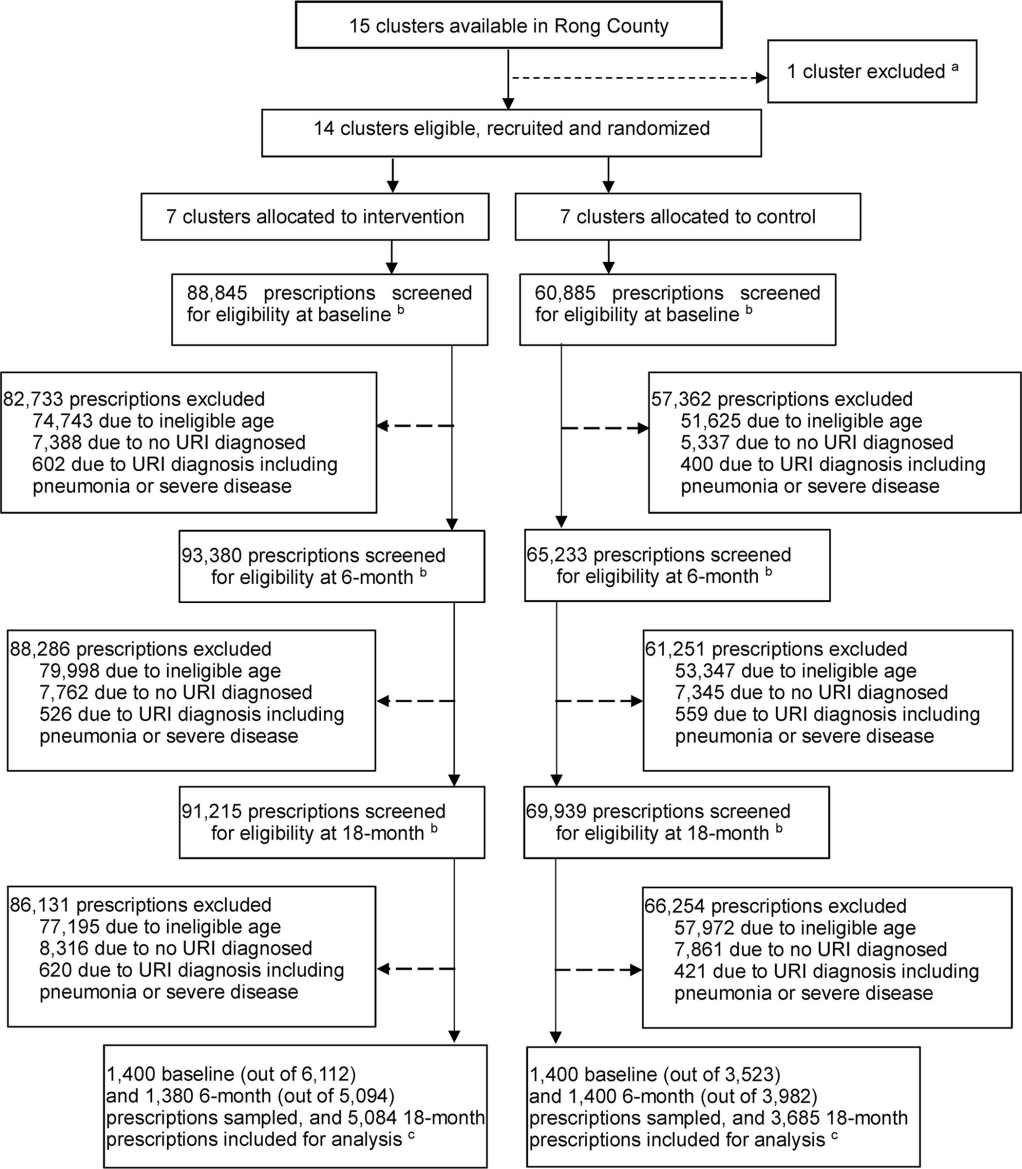
CONSORT trial flow diagram. ^a^We excluded one township hospital located in the county centre because it is not closely comparable due to having much better staff capacity and equipment than its peers and being close to the county general hospital. ^b^Baseline prescriptions were prescribed during the 3 months prior to the start of the 6-month intervention period, 6-month trial endline prescriptions were prescribed during the final 3 months of the 6-month intervention period, and the 18-month follow-up prescriptions were prescribed during the final 3 months of the 18-month period since the intervention was first implemented. ^c^To evaluate the primary and secondary outcomes, we randomly selected 200 eligible prescriptions from each township hospital at both baseline and the 6-month trial endline, or all such prescriptions from township hospitals for which the total number available was less than 200. All eligible prescriptions were included from the 18-month follow-up. In all analyses, prescriptions were analysed according to the treatment arm their township hospital was originally allocated to. Clusters = township hospitals. CONSORT, Consolidated Standards of Reporting Trials; URTI, upper respiratory tract infection.

We followed up all 14 facilities (seven intervention and seven control) in Rong county at 6 and 18 months after the intervention was implemented. At baseline, out of 88,845 prescriptions issued in the intervention arm, 6,112 (6.8%) were eligible for inclusion in the study, and in the control arm, 3,523 out of 60,885 (5.4%) prescriptions were eligible. At 6 months in the intervention arm, 5,094 out of 93,380 (5.5%) prescriptions were eligible, and in the control arm, 3,982 out of 65,233 (6.1%) prescriptions were eligible. At 18 months in the intervention arm, 5,084 out of 91,215 (5.6%) prescriptions were eligible, and in the control arm, 3,685 out of 69,939 (5.3%) prescriptions were eligible (Fig 1). We found that patient and doctor characters were well balanced across the three periods with a modest (>10%) imbalance in payment methods, doctor’s years of work, and diagnosis (acute pharyngitis and laryngitis) (Table 1). Our primary outcome sensitivity analysis that included diagnosis as a covariate indicated no substantive differences from the adjusted analysis excluding diagnosis (S4 Table), and so we did not include diagnosis in any other analyses and only discuss the primary outcome results from the main adjusted analysis. Additionally, for some covariate-adjusted analyses of prescribing outcomes, the preferred GEEs failed to converge, and we had to use less preferred forms of the GEEs (Table 2). However, for all crude analyses, the preferred GEEs converged, and as the results differed little between the covariate-adjusted and crude analyses (S2 Table), the covariate-adjusted results from the less preferred forms of the GEEs appear robust.

**Table 1:** Patient and doctor characteristics at baseline, 6-month follow-up (trial endline), and 18-month follow-up.

	Intervention			Control		
	Baseline	6 months (trial endline)	18 months	Baseline	6 months (trial endline)	18 months
**No. clusters**	7	7	7	7	7	7
**Patients’ characteristics**						
No. of prescriptions	1,400	1,380	5,084	1,400	1,400	3,685
**Sex**						
Male	827 (59%)	779 (56%)	2,962 (58%)	776 (55%)	817 (58%)	2,219 (60%)
Female	572 (41%)	601 (44%)	2,122 (42%)	624 (45%)	583 (42%)	1,466 (40%)
Missing	1 (<1%)	0	0	0	0	0
**Age group**						
2–4	952 (68%)	891 (65%)	3,312 (65%)	884 (63%)	957 (68%)	2,263 (61%)
5–14	448 (32%)	489 (35%)	1,772 (35%)	516 (37%)	443 (32%)	1,422 (39%)
**Diagnoses**						
J00 Acute nasopharyngitis (common cold)	51 (4%)	72 (5%)	414 (8%)	64 (5%)	87 (6%)	400 (11%)
J01 Acute sinusitis	19 (1%)	11 (1%)	175 (3%)	18 (1%)	17 (1%)	11 (<1%)
J02 Acute pharyngitis	465 (33%)	537 (39%)	1,109 (22%)	516 (37%)	522 (37%)	1,471 (40%)
J03 Acute tonsillitis	150 (11%)	121 (9%)	599 (12%)	110 (8%)	138 (10%)	401 (11%)
J04 Acute laryngitis and tracheitis	58 (4%)	0	537 (11%)	0	2 (<1%)	286 (8%)
J05 Acute obstructive laryngitis (croup) and epiglottitis	0	0	0	0	0	0
J06 Acute upper respiratory infections of multiple and unspecified sites	657 (47%)	639 (46%)	2,250 (44%)	692 (49%)	634 (45%)	1,116 (30%)
**Payment method**						
Insurance copayment	1,090 (78%)	970 (70%)	2,584 (51%)	1,013 (72%)	889 (64%)	1,127 (31%)
Fully out of pocket	310 (22%)	410 (30%)	2,500 (50%)	387 (28%)	511 (36%)	2,558 (69%)
No. of medicines prescribed	5.1 (±1.5)	4.8 (±1.3)	4.7 (±1.2)	4.6 (±1.4)	4.8 (±1.4)	4.8 (±1.4)
**Doctor characteristics**						
No. of doctors	79	83	85	88	83	89
**Sex**						
Male	50 (63%)	52 (63%)	53 (62%)	52 (59%)	49 (59%)	53 (60%)
Female	29 (37%)	30 (36%)	31 (37%)	35 (40%)	33 (40%)	35 (39%)
Missing	0	1 (1%)	1 (1%)	1 (1%)	1 (1%)	1 (1%)
**Age group**						
≤35	37 (47%)	37 (45%)	39 (46%)	31 (35%)	30 (37%)	31 (35%)
36–44	35 (44%)	38 (46%)	38 (45%)	41 (47%)	40 (48%)	41 (46%)
≥45	7 (9%)	7 (8%)	7 (8%)	15 (17%)	12 (14%)	16 (18%)
Missing	0	1 (1%)	1 (1%)	1 (1%)	1 (1%)	1 (1%)
**Qualification level**						
3 years medical education	59 (75%)	60 (72%)	61 (72%)	68 (77%)	65 (79%)	70 (79%)
MBBS (5 years)	20 (25%)	22 (27%)	23 (27%)	19 (22%)	17 (20%)	18 (20%)
Missing	0	1 (1%)	1 (1%)	1 (1%)	1 (1%)	1 (1%)
**Years of work**						
≤5	12 (15%)	13 (16%)	13 (15%)	23 (26%)	20 (24%)	24 (27%)
6–10	22 (28%)	22 (26%)	23 (27%)	18 (20%)	17 (20%)	18 (20%)
≥11	44 (57%)	47 (57%)	48 (57%)	46 (53%)	45 (55%)	46 (52%)
Missing	0	1 (1%)	1 (1%)	1 (1%)	1 (1%)	1 (1%)

Data are *n* (%) or mean (±SD).

**Table 2:** Covariate-adjusted intervention effects for outcomes at 6-month follow-up and 18-month follow-up.

Outcome	Period	Intervention^a^	Control^a^	Intervention-control difference at 6-/18-month follow-up versus difference at baseline (95% CI; *P* value)^b^	Intervention-control difference at 18-month follow-up versus difference at 6 months (95% CI; *P* value)^b^
**APR**^**☯**^					
	Baseline	1,171/1,400 (84%)	1,063/1,400 (76%)		
	6 months	515/1,380 (37%)	1,084/1,400 (77%)	−49 pp (−63 to −35); <0.0001	
	18 months	2,748/5,084 (54%)	2,772/3,685 (75%)	−36 pp (−55 to −17); <0.0001	13 pp (−7 to 33); 0.21
**Multiple APR**^¶^					
	Baseline	103/1,171 (9%)	83/1,063 (8%)		
	6 months	29/515 (6%)	65/1,084 (6%)	−2 pp (−7 to 3); 0.51	
	18 months	54/2,748 (2%)	209/2,772 (8%)	−6 pp (−13 to 0); 0.048	−5 pp (−8 to −1); 0.008
**Broad-spectrum APR**^**☯**^					
	Baseline	942/1,171 (80%)	787/1,063 (74%)		
	6 months	346/515 (67%)	794/1,084 (73%)	−12 pp (−21 to −4); 0.005	
	18 months	2,089/2,748 (76%)	2,082/2,772 (75%)	−20 pp (−34 to −6); 0.006	−8 pp (−22 to 6); 0.28
**Infusion APR**^‡^					
	Baseline	252/1,171 (22%)	244/1,063 (23%)		
	6 months	110/515 (21%)	331/1,084 (31%)	−8 pp (−23 to 7); 0.31	
	18 months	266/2,748 (10%)	365/2,772 (13%)	−2 pp (−15 to 11); 0.8	6 pp (−12 to 24); 0.5
**Antiviral prescription rate**^**☯**^					
	Baseline	901/1,400 (64%)	609/1,400 (44%)		
	6 months	942/1,380 (68%)	732/1,400 (52%)	−3 pp (−18 to 11); 0.64	
	18 months	3,017/5,084 (59%)	1,778/3,685 (48%)	−3 pp (−29 to 24); 0.85	1 pp (−21 to 22); 0.93
**Glucocorticoid prescription rate**^¶^					
	Baseline	326/1,400 (23%)	304/1,400 (22%)		
	6 months	298/1,380 (22%)	254/1,400 (18%)	2 pp (−11 to 14); 0.8	
	18 months	1,149/5,084 (23%)	585/3,685 (16%)	3 pp (−10 to 15); 0.69	1 pp (−11 to 13); 0.87
**Vitamin prescription rate**^**☯**^					
	Baseline	192/1,400 (14%)	245/1,400 (18%)		
	6 months	166/1,380 (12%)	223/1,400 (16%)	0 pp (−7 to 7); 0.97	
	18 months	936/5,084 (18%)	700/3,685 (19%)	4 pp (−3 to 11); 0.24	4 pp (−5 to 13); 0.41
**Traditional Chinese medicine prescription rate**^**☯**^					
	Baseline	1,152/1,400 (82%)	999/1,400 (71%)		
	6 months	1,231/1,380 (89%)	1096/1,400 (78%)	−1 pp (−13 to 12); 0.92	
	18 months	4,501/5,084 (89%)	3,077/3,685 (84%)	−2 pp (−12 to 7); 0.66	−1 pp (−8 to 5); 0.66
**Nonantibiotic medicine prescription rate**^**☯**^					
	Baseline	1,218/1,400 (87%)	1,194/1,400 (85%)		
	6 months	1,179/1,380 (85%)	1,215/1,400 (87%)	−2 pp (−8 to 4); 0.52	
	18 months	4,428/5,084 (87%)	3,255/3,685 (88%)	−6 pp (−16 to 3); 0.17	−4 pp (−10 to 1); 0.1
**Full prescription cost (USD)**^¶^					
	Baseline	4.2 (±1.6)	4.4 (±1.9)		
	6 months	4.2 (±1.6)	4.4 (±2.0)	−0.09 (−0.34 to 0.15); 0.46	
	18 months	4.5 (±2.2)	4.7 (±3.0)	0.02 (−0.41 to 0.45); 0.93	0.11 (−0.36 to 0.58); 0.64
**Antibiotics cost (USD)**^¶^					
	Baseline	0.6 (±0.4)	0.5 (±0.4)		
	6 months	0.3 (±0.4)	0.5 (±0.4)	−0.35 (−0.45 to −0.25); <0.0001	
	18 months	0.4 (±0.4)	0.5 (±0.4)	−0.26 (−0.38 to −0.13); <0.0001	0.09 (−0.05 to 0.23); 0.19
**Other medication cost (USD)**^¶^					
	Baseline	2.2 (±1.5)	2.4 (±1.9)		
	6 months	2.5 (±1.5)	3.1 (±2.7)	0.24 (0.01 to 0.48); 0.043	
	18 months	2.7 (±2.1)	2.8 (±2.9)	0.24 (−0.16 to 0.64); 0.24	0.00 (−0.46 to 0.45); 0.99

^a^Intervention and control-arm summary data for prescribing rate outcomes are the number of prescriptions containing the relevant medicine divided by the total number of prescriptions (percentage), and for cost outcomes are mean (±SD).

^b^Estimated treatment effects represent either the difference between the intervention minus control difference at 6 months and the intervention minus control difference at baseline, or the difference between the treatment effect at 18 months and the treatment effect at baseline months, or they represent the difference between the treatment effect at 18 months and the treatment effect at 6 months as indicated, after adjusting for patient sex, age, and insurance payment status, and prescribing doctor sex, age, and education level.

Treatment effects for prescribing rate outcomes are on the absolute pp scale, and for cost outcomes are in USD per prescription, with each prescription representing one patient–doctor consultation. The between-time-period difference in treatment-arm differences (and the associated 95% CIs and *P* values) are estimated by GEE coefficients for the interaction between treatment arm and time period. The GEEs use either binomial errors and an identity link (☯, ‡) for binary prescribing outcomes, or Gaussian errors and an identity link (¶) for continuous cost outcomes or those binary prescribing outcomes where the binomial and then Poisson identity models failed to converge. GEEs accounted for clustering within facilities and within facilities across time periods either using an exchangeable correlation matrix (☯, ¶) or an identity matrix (‡) where the GEEs failed to converge with an exchangeable correlation matrix, given that GEEs are robust to misspecification of the correlation matrix. Outcome data were present for all outcomes and time periods, but <1% of covariate data were missing (see S3 Table). Analyses therefore excluded all patient prescriptions with missing covariate data and assume that data are missing at random. There were no changes to the original allocation of facilities.

**Abbreviations:** APR, antibiotic prescription rate; GEE, generalised estimating equation; pp, percentage points; USD, US dollars.

### Antibiotic prescribing

Between baseline and 6 months, the raw overall APR reduced from 84% to 37% in the intervention arm compared to no substantive change in the control arm (76% to 77%; Table 2). Accounting for the baseline intervention-control difference, and after controlling for potential confounders, there was a 6-month intervention-arm versus control-arm reduction in the APR of −49 pp (95% CI −63 to −35; *P* < 0.0001). At 18 months, the APR was 54% in the intervention arm and 75% in the control arm. Accounting for the treatment-arm difference at baseline, there was a covariate-adjusted 18-month intervention-arm versus control-arm reduction in the APR of −36 pp (95% CI −55 to −17; *P* < 0.0001). Accounting for the treatment-arm difference at 6 months and adjusting for covariates at 18 months, there was no intervention-arm versus control-arm change in the APR: 13 pp (95% CI −7 to 33; *P* = 0.21). Intra-cluster correlation coefficient estimates for the APR outcome are presented in S2 Table.

**Fig 2:**
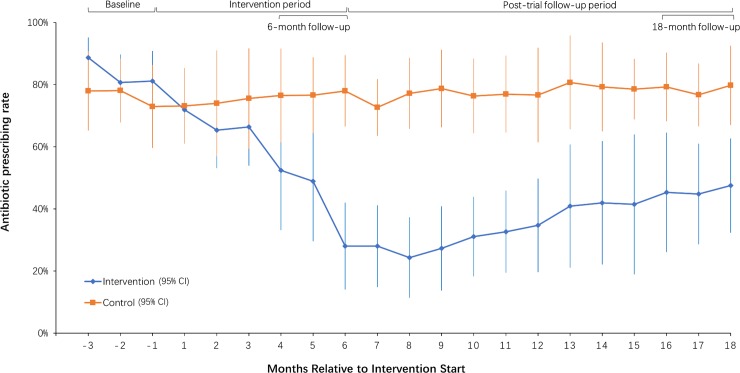
Township hospital monthly APRs (intervention and control arms) for children (2–14 years old) diagnosed with URTIs before, during, and after the intervention period. Data points are monthly mean APRs calculated from cluster-level APR values. Error bars are 95% CIs. APR, antibiotic prescription rate; URTI, upper respiratory tract infection.

**Fig 3:**
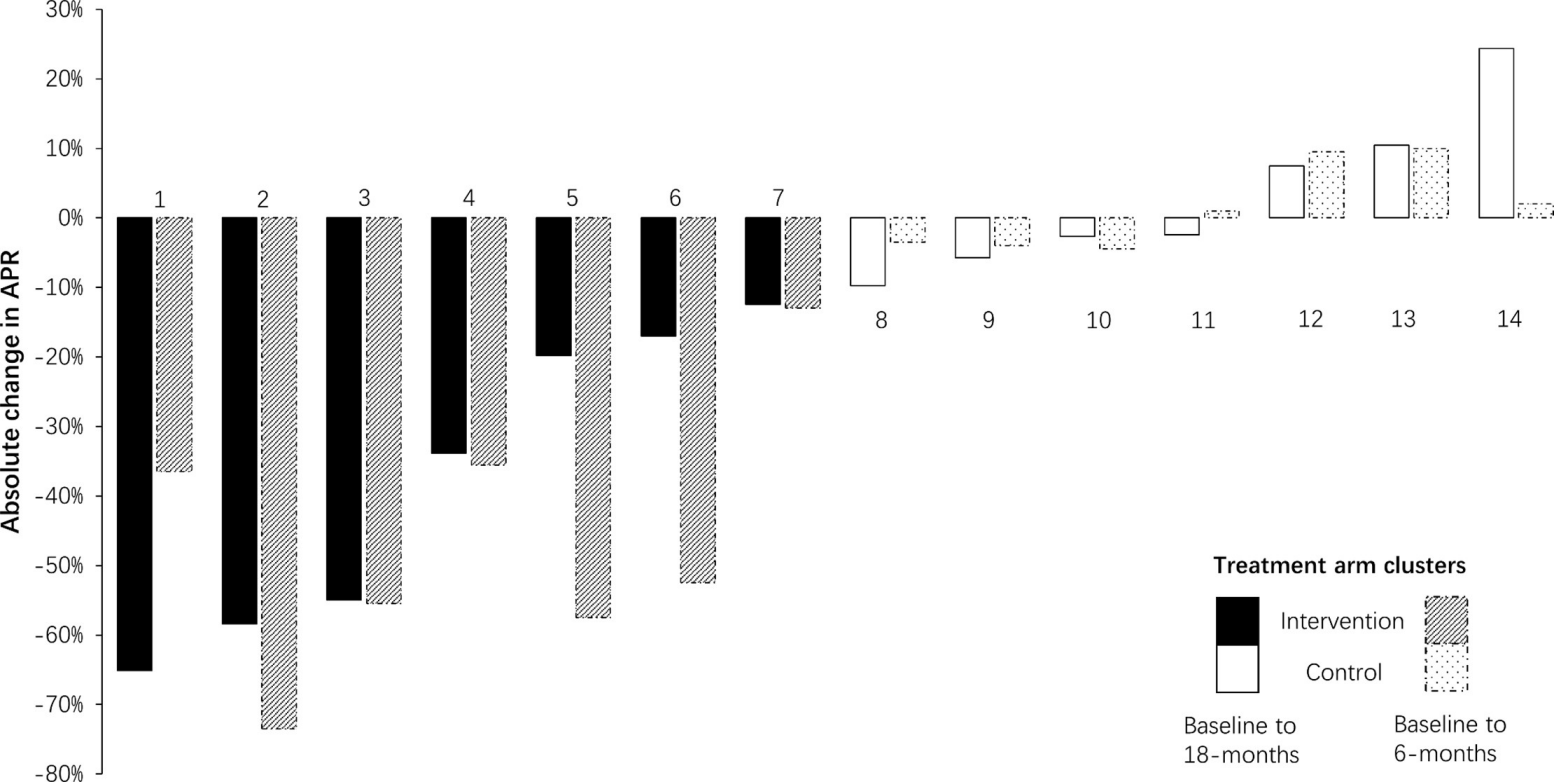
Facility-level changes in the APR for childhood (2–14 years old) URTIs from baseline to 6- and 18-month follow-up. Dotted bars represent changes between baseline and 6 months (trial endline), while solid bars represent changes between baseline and 18-month follow-up (12 months after trial endline). Facility numbers 2, 5 and 8 were included in the qualitative study. APR, antibiotic prescription rate; URTI, upper respiratory tract infection.

Fig 2 shows how the intervention-arm APR declined substantially following the intervention, before plateauing and then appearing to gradually increase between 6 months and 18 months, although the statistical analysis showed no statistically significant change in the APR between 6 and 18 months (Table 2). In the control arm, prescribing was constant throughout at around 80%. Fig 3 shows that there were large variations in APR reductions in intervention facilities at 6 months versus baseline (−74% to −13%) and at 18 months versus baseline (−65% to −12%), compared to much smaller variations in control facilities (−10% to 10% at 6 months versus baseline, and −6% to 10% at 18 months versus baseline). While the intervention effect was sustained in most intervention facilities at 18 months, in one (facility 1) it increased, and in two, it was substantially reduced (facility 5 and 6).

### Secondary prescribing and cost outcomes

Compared to baseline intervention-control differences at 6-month follow-up, there was a modest reduction in the broad-spectrum APR (−12 pp [95% CI −21 to −4]), a large reduction in the cost of antibiotics per prescription (−0.35 US dollars [USD; 95% CI −0.45 to −0.25]), and a modest increase in the cost of all other nonantibiotic medications per prescription (0.24 USD [95% CI 0.01–0.48]) (Table 2). Compared to baseline intervention-control differences at 18-month follow-up, there was a possible small reduction in the multiple APR (−6 pp [95% CI −13 to 0]), a moderate reduction in the broad-spectrum APR (−20 pp [95% CI −34 to −6]), and a large reduction in the cost of antibiotics (−0.26 [95% CI −0.38 to −0.13]). Compared to differences at 6-month and 18-month follow-up, only the multiple APR showed any clear difference between treatment arms, with an additional—but small—possible reduction (−5 pp [95% CI −8 to −1]) (Table 2). We also observed an overall high rate of prescribing for antivirals (around 50%), glucocorticoids (around 20%), and traditional Chinese medicines (around 80%).

### Qualitative study

Doctors in the two selected intervention facilities reported better clinical knowledge in appropriate use of antibiotics and continued to refer to our intervention guidelines in their practice. They reported enhanced communication skills with patients/caregivers, as well as increased confidence in not giving antibiotics for common URTIs, e.g., one doctor reported, “Prescription has to follow patient symptoms and diagnosis [as in the guidelines]. I tell patients [caregivers] with common colds to have a rest without taking any medicines. If they do not trust me, go to find another doctor” (p005; doctor, male, 25–34 years old). In the control facility, doctors (not trained on the clinical guideline and communication skills) reported reasons or excuses for giving antibiotics as stated at baseline [23], such as the difficulty in differentiating between viral and bacterial infections or avoiding patient complaints. We found that peer-review meetings continued in all the intervention facilities, but with variation. The better performing facilities continued to organise monthly peer-review meetings that were led by senior doctors, issued specific targets to limit antibiotic use (e.g., a maximum of 20% APR for outpatients), evaluated individual doctors’ prescription histories, and imposed specific fines (e.g., 0.08 USD per unqualified prescription). In the less well performing facility, peer-review meetings were less frequent (once a quarter) and had less stringent targets (e.g., a maximum of 40% APR for outpatients).

Township directors reported that the lack of further supervisory and monitoring activities by health authorities around antibiotic prescribing had downgraded the importance of the antimicrobial stewardship programme in their perceptions. Caregivers in the intervention facilities reported having received warnings on antibiotic overuse during their consultations, but facilities stopped playing the information videos in waiting rooms and ran out of leaflets. A few patients from both arms reported receiving antibiotics from village doctors or obtaining amoxicillin in private pharmacies without prescriptions (see themes and detailed quotations in S5 Table and associated interview guides in S1 Text).

## Discussion

To our knowledge, our study is the first to investigate the post-trial sustainability of an antimicrobial stewardship programme in a resource-poor setting. Our main finding is that our comprehensive intervention, which targeted doctors and caregivers, was associated with a substantial reduction in antibiotic prescribing 18 months after implementation, or 12 months after all trial-directed intervention activities stopped. At both 6 months (the trial endline) and 18 months (12 months after the trial-directed intervention period) compared to baseline, our intervention achieved a clear reduction in antibiotic prescribing (in the one county for which we had data) that was much higher than what has been reported in previous similar trials (albeit in HIC settings) [8,10]. However, although there was no statistically significant change in antibiotic prescribing between 6 months and 18 months, we would not expect the improvements to be fully sustained over this and longer time periods in the absence of any further intervention-related inputs.

Looking only at those prescriptions where antibiotics were issued, we also observed a small reduction in the relative prescribing rate for multiple antibiotics versus single antibiotics at 18 months compared to treatment-arm differences at baseline, and at 18 months compared to treatment-arm differences at 6 months. Similarly, looking only at those prescriptions where antibiotics were issued, we also observed a moderate reduction in the relative prescribing rate for broad-spectrum antibiotics versus narrow-spectrum antibiotics at 6 months compared to treatment-arm differences at baseline, and at 18 months compared to treatment-arm differences at baseline. However, these relative prescribing rate results for specific types of antibiotics are at risk of bias (see limitations below) in comparison to the US, where a trial that used doctor education reduced the overall rate of broad-spectrum antibiotic prescribing [24].

In interpreting our study’s findings, it should be noted that we only reported results from the 14 facilities in one county (Rong), not from all 25 facilities in our trial, due to logistical constraints in obtaining the paper-based records from the other county (Liujiang). The intervention achieved a larger reduction (point estimate) of APR at 6 months in Rong county alone compared to when we analysed both counties’ 6-month data in our trial endline results paper (−49 pp [95% CI −63 to −35] versus −29 pp [95% CI −42 to −16]). This indicated potential heterogeneity in effectiveness between counties, although the overlap in CIs indicates that this conclusion must be treated cautiously.

Evidence from this long-term follow-up study shows that our intervention appears to have changed doctors’ knowledge and attitudes [23] and led to long-term benefits in antibiotic prescribing. This is surprising given the fact that improved knowledge among rural doctors has often not led to the more rational use of antibiotics [25]. In addition, China’s health reform policies, such as introducing the essential medicine list, a “Zero Mark-Up” policy for medications dispensed in hospital pharmacies, and a target cap of 20% for outpatient antibiotic prescribing did not result in reduced APRs in primary care settings [26–28]. Our success in behaviour change may have been achieved because the intervention was designed to be easily embedded into routine practice in township hospitals. Doctors reported that they maintained better knowledge in antibiotic use, improved communication skills with patients, and had the confidence to not prescribe antibiotics despite caregivers’ requests. This echoes findings from the Netherlands, where enhanced clinician communication skills were found to be the main cause of sustained reductions in antibiotic prescribing [11]. Peer-review meetings seemed to be another important contributing factor. In this study, the better performing facilities continued to conduct monthly peer-reviews strategies, such as prescribing targets, issuing specific prescribing feedback, and small fines for doctors who overprescribed. These factors were also highlighted in a qualitative systematic review [29] as key to changing doctors’ antibiotic prescribing behaviours. Our antimicrobial stewardship programme has also been proved cost-effective: implementation costs 391 USD per facility, and the incremental cost is 0.03 USD per pp reduction in APR at 6 months [30]. We also observed a reduction of infusion antibiotics in both arms, which may be due to the introduction of regulations to reduce infusions by the County Health Bureau during our study period.

However, we believe that refresher training sessions and regular supervisory and monitoring should be continued if the intervention effect is to be sustained beyond 18 months. Reduction in effect is common after behaviour change interventions. In a trial in Vietnam, though antibiotic prescribing was reduced by 14 pp after C-reactive protein tests had been introduced to rural primary care facilities, the positive effect ceased soon after the study because of patient demands, incentives provided by pharmaceutical companies, and the pressure to replenish pharmacy stocks in these facilities [14]. In our study, only two intervention facilities exhibited a substantial reduction of intervention effect on the APR at 18 months, which may have been caused by factors such as a change of hospital leadership, while all other intervention facilities exhibited little change from 6 to 18 months.

Though we did not observe any clear increase in prescribing of other medications as a substitution effect for reducing antibiotic prescribing, the overall high prescribing rates for antivirals, glucocorticoids, and traditional Chinese medicines were likely to be inappropriate. This may be related to doctors’ lack of knowledge and/or the pressures of maintaining prescription revenues (from health insurance and/or out-of-pocket revenue). Glucocorticoid steroids may be beneficial to asthma but not to URTIs. Occasional use of antivirals such as oseltamivir may be justified in high-risk children if symptoms suggest influenza. However, the generally high rate of antiviral prescribing is likely to be inappropriate in children with URTIs [31]. Patient preference and requests for symptom relief may lead to the very high prescription rate (80%) of traditional Chinese medicines observed. Another possible explanation is that township hospitals have financial incentives to prescribe traditional Chinese medicines because they are exempt from the Zero Mark-up policy, and so they can add a 15% or more margin when dispensed from hospital pharmacies. We also observed a reduction of infusion antibiotics in both arms at 18 months, which may be due to the introduction of regulations to reduce infusions by the County Health Bureau during our study period.

Our study has several limitations. First, we did not include all facilities from the original trial, but only in one county for which there was a higher effect size than in the other county. The overall effect may be less across the two counties and if scaled-up more widely. Second, we did not collect actual antibiotic consumption. China has strengthened its antibiotic stewardship policy since 2012 to restrict antibiotic prescription to qualified physicians, while village doctors are considered restricted practitioners who do not have the right to prescribe antibiotics. In practice, we found gaps in enforcement, with some patients reporting obtaining antibiotics from village doctors or private pharmacies. Future interventions should include village doctors, given that they act as the first contact for medical consultations in rural areas [25], and utilise innovative ways to measure antibiotic consumption. Third, our secondary outcomes measuring the prescribing rates of specific types of antibiotics were relative rates with the denominator being all prescriptions containing one or more antibiotics. Because these are outcomes were effectively measured for subgroups that were determined post randomisation and were conditional on antibiotic prescription, there is a risk of bias/confounding, and these results should therefore be treated cautiously. Fourth, we did not measure harms from the trial, such as return visits or sepsis cases. However, doctor educational or shared decision-making interventions have not previously increased severe bacterial infections or reconsultations [32]. Fifth, the facilities in which we performed qualitative interviews were preselected based on the trial results, restricting our ability to collect information regarding the reasons why two facilities relapsed in prescribing rates during post-trial follow-up. Sixth, the study was conducted in rural southwest China, where findings may not be easily generalisable to other low-resource settings because health systems may be different. However, interventions in this study targeted similar real-world challenges in other LMICs, including widespread inappropriate use of antibiotics for URTIs, lack of knowledge on antimicrobial resistance among health providers, lack of antimicrobial resistance stewardship programmes, and demands for antibiotics from patients.

## Conclusion

We found that, in one county of the trial sites, the benefits of our antimicrobial stewardship programme on antibiotic prescribing were well maintained 18 months after implementation, or 12 months after trial-led activities finished. This indicates that effects may be sustained for interventions including evidence-based guidelines, peer-review meetings, improved doctor–patient communications, and the provision of concise education to caregivers during consultations, in similar settings. However, although there was no statistically significant change in the APR after trial activities ceased, it is of course expected that, without further intervention-related inputs, the improvements in antibiotic prescribing would decline. This study implies that this type of intervention may be successful in other LMICs with similar challenges.

## Supporting information

S1 CONSORT Checklist (DOCX)

S1 Text Interview guides for qualitative study. (DOCX)

S1 Data Aggregated outcome data by cluster at baseline, 6 months, and 18 months after randomisation. (XLSX)

S1 Table ICD-10 codes used to classify URTIs from prescription data. ICD, International Classification of Diseases; URTI, upper respiratory tract infection.
(DOCX)

S2 Table Crude intervention effects on prescriptions of antibiotic and other medications, and costs for 6-month and 18-month follow-up. (DOCX)

S3 Table Missing data frequency (including outcome and all covariates) for all analyses. (DOCX)

S4 Table Covariate-adjusted intervention effects for the primary outcome at 6-month and 18-month follow-up: A sensitivity analysis including diagnosis. (DOCX)

S5 Table Key themes and examples of evidence in the qualitative study. (DOCX)

## Abbreviations

APR: antibiotic prescription rate
CDC: Centre for Disease Control and Prevention
CONSORT: Consolidated Standards of Reporting Trials
cRCT: cluster-randomised controlled trial
GEE: generalised estimating equation
HIC: high-income country
LMIC: low- and middle-income country
pp: percentage points
TDF: theoretical domains framework
URTI: upper respiratory tract infection
USD: US dollars
