# Supplementary material for: Long-term outcomes of an educational intervention to reduce antibiotic prescribing for childhood upper respiratory tract infections in rural China: Follow-up of a cluster-randomised controlled trial
Source: PLoS Med. 2019 Feb 5;16(2):e1002733. doi: 10.1371/journal.pmed.1002733 (PMC6363140; doi:10.1371/journal.pmed.1002733)
Supplement: S1 Text — (DOCX) [file pmed.1002733.s002.docx]

**S6 Text. Interview Guides for Qualitative Study.**

**Interview guide for intervention arm**

*Hospital director*

- Is there any new antibiotic use/ control policy after the trial ended? If yes, how were they implemented? What do you think about these policies?
- Is there any change on purchasing system in the hospital, patient reimbursement and doctor salary determinants after the trial ended?
- Is there any action for antibiotic control in your hospital? If yes, what are they? Is there any difference compared with the trial period?
- Are there any new administrative regulations regarding antimicrobial resistance stewardship (AMS) in the last year? How they affected your daily activity?
- Are the desk guides of Childhood fever and URI management used in practice?
- Is there any intervention activity still being conducted after the trial ended? Why or why not? If yes, how were they implemented? (Training? Using desk guides of Childhood fever and URTI management? Peer review meeting? Delivering health education messages during health consultation? Health education leaflets on antibiotics use? Health education video on antibiotics use?) If the peer review of antibiotics prescribing is still conducted in routing meeting, what have being done in this meeting? Frequency of the meeting? Actions on high antibiotics prescribing rate?
- Give special attention to peer review meetings regarding any goals to be achieved, who led the monitoring and discussion, any specific feedbacks, and actions for high prescribers?

*Doctor*

- Is a consultation always ends with prescriptions? What conditions should be given an antibiotic and what conditions not?
- Did you attend any training regarding antibiotics use after the trial ends? (If yes, the frequency? Content?)
- Are you still using the desk guides/guideline of Childhood fever and URTI management? Why or why not?
- Did you attend any peer review meeting after the trial ended? (If yes, the frequency? Content? Consequence of high APR?) Give special attention to peer review meetings regarding any goals to be achieved, who led the monitoring and discussion, any specific feedbacks, and actions for high prescribers?
- Are you still providing health education on antibiotics use for caregivers of childhood URTI patients? Why or why not?
- Is there any difference of your standard of prescribing antibiotics (in what situation you will decide to prescribe antibiotics) for childhood URTI compared with trial period? If yes, what made that difference happen? If not, what made you keep that practice?

*Caregiver*

- How do you know about antibiotics? Where did you get the information about antibiotics usually?
- Have you watched the health education video played in the hospital and do you understand it?
- Do you understand what you were told in the consultation?
- Do you know what to do if the child is sick with a common cold?
- Did the doctor explain what you should do if your child is sick with a common cold?
- Do you understand what an antibiotic is and when to use it?
- If your child has a common cold but is not prescribed an antibiotic, how does that make you feel/ what do you think?
- Do you believe that children can get better without an antibiotic?
- Do you get an antibiotic through another means e.g. village doctor, pharmacy? Why or why not?

**Interview guide for control arm**

*Hospital director*

- Is there any new antibiotic use/ control policies after the trial ended? If yes, how are they implemented? What do you think about these policies?
- Is there any change on purchasing system in the hospital, since the trial ended?
- Is there any change in the patient reimbursement system, since the trial ended?
- Is there any change in the doctor salary determinants after the trial ended?
- What are your current stewardship practices of antibiotics prescribing in your township hospital? Is there any difference compared with the trial period? (Specifically is there any policy, actions from the health bureau on use of antibiotics?)

*Doctor*

- What do you prescribe for a URTI case? Why?
- Is there any difference of your standard of prescribing antibiotics (in what situation you will decide to prescribe antibiotics) for childhood URTI compared with the trial period? If yes, what made that difference happen?

*Caregiver*

- How do you know about antibiotics? Where did you get the information about antibiotics usually?
- Are there any materials for antibiotic use in township hospitals, such as videos or pamphlets? If yes, how do you think the usefulness?
- Do you understand what you were told in the consultation?
- Do you know what to do if the child is sick with a common cold?
- Did the doctor explain what you should do if your child is sick with a common cold?
- Do you understand what an antibiotic is and when to use it?
- If your child has a common cold but is not prescribed an antibiotic, how does that make you feel/ what do you think?
- Do you believe that children can get better without an antibiotic?
- Do you get an antibiotic through another means e.g. village doctor, pharmacy? Why or why not?
