# Supplementary material for: Long-term outcomes of an educational intervention to reduce antibiotic prescribing for childhood upper respiratory tract infections in rural China: Follow-up of a cluster-randomised controlled trial
Source: PLoS Med. 2019 Feb 5;16(2):e1002733. doi: 10.1371/journal.pmed.1002733 (PMC6363140; doi:10.1371/journal.pmed.1002733)
Supplement: S1 Table — ICD, International Classification of Diseases; URTI, upper respiratory tract infection. (DOCX) [file pmed.1002733.s004.docx]

**S1 Table. ICD-10 codes used to classify upper respiratory tract infections from prescription data.**

| **ICD-10 codes** | **Diagnosis** |
| --- | --- |
| **URTIs included** | |
| J00 | Acute nasopharyngitis (common cold) |
| J01 | Acute sinusitis |
| J02 | Acute pharyngitis |
| J03 | Acute tonsillitis |
| J04 | Acute laryngitis and tracheitis |
| J05 | Acute obstructive laryngitis (croup) and epiglottitis |
| J06 | Acute upper respiratory infections of multiple and unspecified sites |
| **Secondary diagnoses excluded** | |
| A15-A19 | Tuberculosis |
| B20-B24 | Human immunodeficiency virus (HIV) disease |
| J20-J22 | Other acute lower respiratory infections |
| J40-J47 | Chronic lower respiratory diseases |
| N18 | Chronic kidney disease |
| **Additional diagnoses excluded (based on using key words as ICD codes were not fully applied in primary care facilities in the research setting)** | |
|  | Pneumonia |
|  | Otitis media |
|  | Cancer |
|  | Suppurative infection |
|  | Congenital heart diseases |
|  | Cutaneous infection |
|  | Urinary tract infection |
|  | Trauma |
|  | Bacterial enteritis |
| URTIs = upper respiratory tract infections.  We applied ICD-10 codes with the inclusion and exclusion criteria when possible. For diagnoses not made according to the ICD-10 codes, we examined keywords of diagnoses commonly used in the local setting. | |
