# Supplementary material for: Long-term outcomes of an educational intervention to reduce antibiotic prescribing for childhood upper respiratory tract infections in rural China: Follow-up of a cluster-randomised controlled trial
Source: PLoS Med. 2019 Feb 5;16(2):e1002733. doi: 10.1371/journal.pmed.1002733 (PMC6363140; doi:10.1371/journal.pmed.1002733)
Supplement: S2 Table — (DOCX) [file pmed.1002733.s005.docx]

| **S2 Table. Crude intervention effects on prescriptions of antibiotic and other medications, and costs for 6-month and 18-month follow-up.** | | | | | |
| --- | --- | --- | --- | --- | --- |
| **Outcome** | **Period** | **Intervention^a^** | **Control^a^** | **Intervention-control difference at 6-/18-month follow-up vs difference at baseline (95% CI; P-value)^b^** | **Intervention-control difference at 18-month follow-up vs difference at 6 months (95% CI; P-value)^b^** |
| **Antibiotic prescription rate^c^** | Baseline | 1171/1400 (84%) | 1063/1400 (76%) |  |  |
|  | 6 months | 515/1380 (37%) | 1084/1400 (77%) | -48pp (-63, -33); <0.0001 |  |
|  | 18 months | 2748/5084 (54%) | 2772/3685 (75%) | -35pp (-55, -16); <0.0001 | 12pp (-7, 32); 0.22 |
| **Multiple antibiotic prescription rate^c^** | Baseline | 103/1171 (9%) | 83/1063 (8%) |  |  |
|  | 6 months | 29/515 (6%) | 65/1084 (6%) | -2pp (-7, 3); 0.54 |  |
|  | 18 months | 54/2748 (2%) | 209/2772 (8%) | -6pp (-12, 1); 0.074 | -4pp (-8, -1); 0.016 |
| **Broad-spectrum antibiotic prescription rate^c^** | Baseline | 942/1171 (80%) | 787/1063 (74%) |  |  |
|  | 6 months | 346/515 (67%) | 794/1084 (73%) | -12pp (-21, -3); 0.008 |  |
|  | 18 months | 2089/2748 (76%) | 2082/2772 (75%) | -19pp (-34, -5); 0.009 | -7pp (-21, 7); 0.3 |
| **Infusion antibiotic prescription rate^c^** | Baseline | 252/1171 (22%) | 244/1063 (23%) |  |  |
|  | 6 months | 110/515 (21%) | 331/1084 (31%) | -8pp (-22, 7); 0.29 |  |
|  | 18 months | 266/2748 (10%) | 365/2772 (13%) | -4pp (-21, 13); 0.65 | 4pp (-15, 22); 0.69 |
| **Antiviral prescription rate^c^** | Baseline | 901/1400 (64%) | 609/1400 (44%) |  |  |
|  | 6 months | 942/1380 (68%) | 732/1400 (52%) | -5pp (-19, 9); 0.49 |  |
|  | 18 months | 3017/5084 (59%) | 1778/3685 (48%) | -3pp (-29, 23); 0.81 | 2pp (-19, 23); 0.87 |
| **Glucocorticoid prescription rates^c^** | Baseline | 326/1400 (23%) | 304/1400 (22%) |  |  |
|  | 6 months | 298/1380 (22%) | 254/1400 (18%) | 2pp (-10, 15); 0.74 |  |
|  | 18 months | 1149/5084 (23%) | 585/3685 (16%) | 2pp (-11, 15); 0.74 | 0pp (-11, 12); 0.98 |
| **Vitamin prescription rate^c^** | Baseline | 192/1400 (14%) | 245/1400 (18%) |  |  |
|  | 6 months | 166/1380 (12%) | 223/1400 (16%) | 0pp (-7, 7); 0.98 |  |
|  | 18 months | 936/5084 (18%) | 700/3685 (19%) | 5pp (-3, 13); 0.25 | 5pp (-5, 15); 0.35 |
| **Traditional Chinese medicine prescription rate^c^** | Baseline | 1152/1400 (82%) | 999/1400 (71%) |  |  |
|  | 6 months | 1231/1380 (89%) | 1096/1400 (78%) | 0pp (-13, 13); 1 |  |
|  | 18 months | 4501/5084 (89%) | 3077/3685 (84%) | -2pp (-12, 8); 0.77 | -2pp (-8, 5); 0.65 |
| **Non-antibiotic medicine prescription rate^c^** | Baseline | 1218/1400 (87%) | 1194/1400 (85%) |  |  |
|  | 6 months | 1179/1380 (85%) | 1215/1400 (87%) | -3pp (-10, 3); 0.34 |  |
|  | 18 months | 4428/5084 (87%) | 3255/3685 (88%) | -7pp (-17, 3); 0.17 | -4pp (-10, 3); 0.25 |
| **Full prescription cost (USD)^d^** | Baseline | 4.2 (±1.6) | 4.4 (±1.9) |  |  |
|  | 6 months | 4.2 (±1.6) | 4.4 (±2.0) | -0.02 (-0.29, 0.25); 0.88 |  |
|  | 18 months | 4.5 (±2.2) | 4.7 (±3.0) | 0.06 (-0.48, 0.59); 0.83 | 0.08 (-0.49, 0.65); 0.79 |
| **Antibiotics cost (USD)^d^** | Baseline | 0.6 (±0.4) | 0.5 (±0.4) |  |  |
|  | 6 months | 0.3 (±0.4) | 0.5 (±0.4) | -0.34 (-0.45, -0.24); <0.0001 |  |
|  | 18 months | 0.4 (±0.4) | 0.5 (±0.4) | -0.26 (-0.39, -0.12); <0.0001 | 0.09 (-0.05, 0.23); 0.22 |
| **Other medication cost (USD)^d^** | Baseline | 2.2 (±1.5) | 2.4 (±1.9) |  |  |
|  | 6 months | 2.5 (±1.5) | 3.1 (±2.7) | 0.31 (0.06, 0.56); 0.017 |  |
|  | 18 months | 2.7 (±2.1) | 2.8 (±2.9) | 0.28 (-0.24, 0.79); 0.29 | -0.03 (-0.6, 0.54); 0.91 |
| ^a^ Intervention and control arm summary data for prescribing rate outcomes are the number of prescriptions containing the relevant medicine/total number of prescriptions (%), and for cost outcomes are mean (±SD). ^b^ Estimated treatment effects represent either the difference between the intervention minus control difference at 6 months and the intervention minus control difference at baseline, or the difference between the treatment effect at 18 months and the treatment effect at baseline months, or the difference between the treatment effect at 18 months and the treatment effect at 6 months as indicated, with no adjustment for any other covariates (crude estimates). Treatment effects for prescribing rate outcomes are on the absolute percentage point (pp) scale, and for cost outcomes are in USD per prescription, with each prescription representing one patient-doctor consultation. The between-time-period difference in treatment arm differences (and the associated 95% confidence intervals and p-values) are estimated by generalised estimating equation (GEE) coefficients for the interaction between treatment arm and time period. The GEEs use either binomial errors and an identity link (^c^) for binary prescribing outcomes, or Gaussian errors and an identity link (^d^) for continuous cost outcomes. All GEEs accounted for clustering within facilities and within-facilities across-time periods either using an exchangeable correlation matrix. There were no missing data. There were no changes to the original allocation of facilities. Unadjusted intracluster correlation coefficient estimates and 95% CIs (based on the variance components from a one-way ANOVA) for the primary outcome (APR): baseline intervention = 0.04 (0.01, 0.18), baseline control = 0.09 (0.04, 0.33), baseline overall = 0.04 (0.01, 0.18); 6 months intervention = 0.09 (0.03, 0.32), 6 months control = 0.1 (0.04, 0.37), 6 months overall = 0.25 (0.14, 0.46); 18 months intervention = 0.14 (0.06, 0.44), 18 months control = 0.1 (0.04, 0.36), 18 months overall = 0.16 (0.09, 0.33). | | | | | |
