# Supplementary material for: Long-term outcomes of an educational intervention to reduce antibiotic prescribing for childhood upper respiratory tract infections in rural China: Follow-up of a cluster-randomised controlled trial
Source: PLoS Med. 2019 Feb 5;16(2):e1002733. doi: 10.1371/journal.pmed.1002733 (PMC6363140; doi:10.1371/journal.pmed.1002733)
Supplement: S3 Table — (DOCX) [file pmed.1002733.s006.docx]

| **S3 Table. Missing data frequency (including outcome and all covariates) for all analyses.** | | | |
| --- | --- | --- | --- |
| **Outcome** | **Period** | **Intervention** | **Control** |
| **Antibiotic prescription rate** | Baseline | 1388/1400 (99.1%) | 1391/1400 (99.4%) |
|  | 6 months | 1372/1380 (99.4%) | 1400/1400 (100%) |
|  | 18 months | 5084/5084 (100%) | 3646/3685 (98.9%) |
| **Multiple antibiotic prescription rate** | Baseline | 1160/1171 (99.1%) | 1054/1063 (99.2%) |
|  | 6 months | 510/515 (99.1%) | 1084/1084 (100%) |
|  | 18 months | 2748/2748 (100%) | 2739/2772 (98.8%) |
| **Broad-spectrum antibiotic prescription rate** | Baseline | 1160/1171 (99.1%) | 1054/1063 (99.2%) |
|  | 6 months | 510/515 (99.1%) | 1084/1084 (100%) |
|  | 18 months | 2748/2748 (100%) | 2739/2772 (98.8%) |
| **Infusion antibiotic prescription rate** | Baseline | 1160/1171 (99.1%) | 1054/1063 (99.2%) |
|  | 6 months | 510/515 (99.1%) | 1084/1084 (100%) |
|  | 18 months | 2748/2748 (100%) | 2739/2772 (98.8%) |
| **Antiviral prescription rate** | Baseline | 1388/1400 (99.1%) | 1391/1400 (99.4%) |
|  | 6 months | 1372/1380 (99.4%) | 1400/1400 (100%) |
|  | 18 months | 5084/5084 (100%) | 3646/3685 (98.9%) |
| **Glucocorticoid prescription rates** | Baseline | 1388/1400 (99.1%) | 1391/1400 (99.4%) |
|  | 6 months | 1372/1380 (99.4%) | 1400/1400 (100%) |
|  | 18 months | 5084/5084 (100%) | 3646/3685 (98.9%) |
| **Vitamin prescription rate** | Baseline | 1388/1400 (99.1%) | 1391/1400 (99.4%) |
|  | 6 months | 1372/1380 (99.4%) | 1400/1400 (100%) |
|  | 18 months | 5084/5084 (100%) | 3646/3685 (98.9%) |
| **Traditional Chinese medicine prescription rate** | Baseline | 1388/1400 (99.1%) | 1391/1400 (99.4%) |
|  | 6 months | 1372/1380 (99.4%) | 1400/1400 (100%) |
|  | 18 months | 5084/5084 (100%) | 3646/3685 (98.9%) |
| **Non-antibiotic medicine prescription rate** | Baseline | 1388/1400 (99.1%) | 1391/1400 (99.4%) |
|  | 6 months | 1372/1380 (99.4%) | 1400/1400 (100%) |
|  | 18 months | 5084/5084 (100%) | 3646/3685 (98.9%) |
| **Full prescription cost (USD)** | Baseline | 1388/1400 (99.1%) | 1391/1400 (99.4%) |
|  | 6 months | 1372/1380 (99.4%) | 1400/1400 (100%) |
|  | 18 months | 5084/5084 (100%) | 3646/3685 (98.9%) |
| **Antibiotics cost (USD)** | Baseline | 1388/1400 (99.1%) | 1391/1400 (99.4%) |
|  | 6 months | 1372/1380 (99.4%) | 1400/1400 (100%) |
|  | 18 months | 5084/5084 (100%) | 3646/3685 (98.9%) |
| **Other medication cost (USD)** | Baseline | 1388/1400 (99.1%) | 1391/1400 (99.4%) |
|  | 6 months | 1372/1380 (99.4%) | 1400/1400 (100%) |
|  | 18 months | 5084/5084 (100%) | 3646/3685 (98.9%) |
| Data are the number of prescriptions where the relevant outcome and all covariate data were available for the model / the total number of prescriptions available (%). For a prescription to be included in an analysis covariate data had to be available on patients’ sex, age, and payment type (insured or fully out-of-pocket); and doctors’ sex, age, and qualification level (based on 3 years of education or 5 years [MBBS equivalent]). | | | |
