# Supplementary material for: Long-term outcomes of an educational intervention to reduce antibiotic prescribing for childhood upper respiratory tract infections in rural China: Follow-up of a cluster-randomised controlled trial
Source: PLoS Med. 2019 Feb 5;16(2):e1002733. doi: 10.1371/journal.pmed.1002733 (PMC6363140; doi:10.1371/journal.pmed.1002733)
Supplement: S4 Table — (DOCX) [file pmed.1002733.s007.docx]

| **S4 Table. Covariate adjusted intervention effects for the primary outcome at 6-month and 18-month follow-up: a sensitivity analysis including diagnosis.** | | | | | |
| --- | --- | --- | --- | --- | --- |
| **Outcome** | **Period** | **Intervention** | **Control** | **Intervention - control difference for 6 months/18 months follow-up vs baseline (95% CI; P-value)^a^** | **Intervention - control difference for 18 months follow-up vs 6 months (95% CI; P-value)^a^** |
| **Antibiotic prescription rate** | Baseline | 1171/1400 (84%) | 1063/1400 (76%) |  |  |
|  | 6 months | 515/1380 (37%) | 1084/1400 (77%) | -47 (-64, -30); <0.0001 |  |
|  | 18 months | 2748/5084 (54%) | 2772/3685 (75%) | -31 (-49, -12); <0.002 | 17 (-1, 34); 0.065 |
| Sensitivity analysis of the covariate adjusted analysis of the primary outcome, including diagnosis in the model given the relatively large imbalances between treatment arms in some diagnostic codes between treatment arms at 18 months. The diagnosis variable was coded as follows: 1) J00 (Acute nasopharyngitis [common cold]), J01 (Acute sinusitis) and J04 (Acute laryngitis and tracheitis), 2) J02 (Acute pharyngitis), 3) J03 (Acute tonsillitis) and 4) J06 (Acute upper respiratory infections of multiple and unspecified sites). Intervention and control arm summary data for prescribing outcomes are number of prescriptions containing the relevant medicine/total number of prescriptions (%), and for cost outcomes mean (±SD). Estimated intervention minus control differences are absolute differences (risk differences) for the relevant time period (6-month or 18-month follow-up) compared to the intervention minus control difference at either baseline or 6-month follow-up as indicated, adjusted for patient sex, age, insurance payment status and diagnosis, and prescribing doctor sex, age and education level. Estimates are calculated using generalised linear models with Gaussian errors and an identity link as binomial and Poisson identity models failed, with the between-time-period difference estimates calculated from the interaction between treatment arm and treatment period. Models were estimated via generalised estimating equations using an exchangeable correlation matrix to account for clustering within facilities and within-facilities across-time periods. Outcome data were present for all outcomes and time periods, but <1% of covariate data were missing (see Table S3 – no additional data were missing in this sensitivity analysis compared to the original adjusted analysis of the primary outcome). Analyses therefore excluded all patient prescriptions with missing covariate data and assume that data are missing at random. There were no changes to the original allocation of facilities. | | | | | |
