# Supplementary material for: Long-term outcomes of an educational intervention to reduce antibiotic prescribing for childhood upper respiratory tract infections in rural China: Follow-up of a cluster-randomised controlled trial
Source: PLoS Med. 2019 Feb 5;16(2):e1002733. doi: 10.1371/journal.pmed.1002733 (PMC6363140; doi:10.1371/journal.pmed.1002733)
Supplement: S5 Table — (DOCX) [file pmed.1002733.s008.docx]

**S5 Table. Key themes and example of evidence in the qualitative study.**

| **Theme** | **Subtheme** | **Example of Evidence** |
| --- | --- | --- |
| Procedural Factors | Use of the guideline | *“We still use the guideline. I think it has changed us a lot. [With the guideline] we know that antibiotics should be contained… We still use it in our routine consultation… It is short, and easy to refer to when needed. I am now more confident to say no if patient requests [antibiotics]” [p001, male, hospital director, 35-44 years old]*  *“Yes, I often use it… mostly during my clinical consultations. If someone has low fever and running nose, I do not give antibiotics.” [p007, male, doctor, 35-44 years old]*  *“I think antibiotics need to be given to elders with acute pharyngitis… it prevents them having a bacterial infection, which is easy for them to get…” [p008, male, doctor, 55+ years old]* |
|  | Prescription review meetings | *“We continued [the prescription review meeting] as before. We do this review for every doctor… I think the first training [of the guideline] was important, but more important is that we have established an AMR stewardship program to review prescriptions regularly. We continued to give a small fine to over-prescribers.” [p001, male, hospital director, 35-44 years old]*  *“We did the review every month. Doctors who had the highest prescription rate are fined for USD$0.06/RMB 0.5 per prescription… Not good for them… We had a policy to achieve less than 20% APR for outpatients … I do not find it difficult.” [p002, female, hospital director, 35-44 years old]*  *“We held less frequent prescription review meetings [compared with the trial period]. We changed a director. He did not do things as the previous director…Sometime he missed the meetings… There was no target set [for APR] now.” [p005, male, doctor, 25-34 years old]*  *“We had a target of 40% APR for outpatients… It is not realistic for doctors to achieve [given the current high APR level].” [p003, male, hospital director, 44-45 years old]* |
|  | Supervision trips | *“We used to held our own training using the guidelines. But we did not do this now. The visit from county level staff helped. We used to have them quite often. But now our hospital director paid less attention to this work [AMR stewardship].” [p004, female, doctor, 25-34 years old]*  *“The County Centre for Disease Control did not visit us any more. It [the visit] was a good learning experience…” [p002, female, hospital director, 35-44 years old]* |
| Inter-personal Factors | Caregiver education | *“I heard about the problem of antibiotics… I heard from the doctor… during my visit to him… Yes, I have seen the pamphlet, but not the video. The pamphlet was useful…” [p011, female, caregiver, 25-34 years old]*  *“I did not see the video. But I heard from my visit to the doctor [regarding the harm of antibiotic overuse].” [p013, female, caregiver, 55+ years old]* |
|  | Caregiver-doctor dynamic | *“I trust the doctor. He told me that using [antibiotics] is not good for my grandson.” [p012, female, caregiver, 55+ years old]*  *“I listen to the doctor. But I will go to a different hospital if my son does not recover in two days.” [p014, male, caregiver, 25-34 years old]*  *“I had to give antibiotics because patients ask for them. They think taking antibiotics leads to a quick cue. Patients will not listen to me...” [p009, male, doctor, 55+ years old]* |
| Contextual Factors | Time constraints | *“Most patients visit us in the morning. I have to deal with each one within 5 minutes… It is hard to find time to explain to them [the harm of using antibiotics].” [p008, male, doctor, 55+ years old]* |
|  | Competition | *“We have to keep patients here. We lose money from the health insurance if we have less patients. We have to satisfy our patients.” [p003, male, hospital director, 44-54 years old]* |
|  | Community influence | *“I often take the left-overs [medications] if someone in the house is sick… I also go to the village doctor… sometime [the village doctor] gives me antibiotics. But I did not give antibiotics to my kids as it was harmful to them… It was OK as I am an adult.” [p016, female, caregiver, 25-44 years old]*  *“Once I visited the [private pharmacy] in the township. I bought some amoxicillin capsules… She [the pharmacist] said it was good for my flu.” [p015, male, caregiver, 55+ years old]* |
